# Health-related quality of life and flourishing in current and former recreational and elite cricketers

**DOI:** 10.1186/s12955-020-01301-7

**Published:** 2020-02-24

**Authors:** Garrett S. Bullock, Gary S. Collins, Nick Peirce, Nigel K. Arden, Stephanie R. Filbay

**Affiliations:** 1 Centre for Sport, Exercise and Osteoarthritis Research Versus Arthritis, University of Oxford, Oxford, UK; 2 Nuffield Department of Orthopaedics, Rheumatology, and Musculoskeletal Sciences, University of Oxford, Oxford, B4495 UK; 3 Centre for Statistics in Medicine, Nuffield Department of Orthopaedics, Rheumatology, and Musculoskeletal Sciences, University of Oxford, Oxford, UK; 4 Oxford University Hospitals NHS Foundation Trust, Oxford, UK; 5 England and Wales Cricket Board, National Cricket Performance Centre, Loughborough University, Loughborough, LE11 3TU UK; 6 National Centre for Sport and Exercise Medicine, Loughborough University, Loughborough, LE11 3TU UK

**Keywords:** Retired athletes, Mental health, Psychological, Resilience, Recreational sport

## Abstract

**Background:**

Health related quality of life (HRQoL) and flourishing are constructs that encompasses a holistic representation of physical, psychological, and social health. The underlying psychological factors that can affect HRQoL and flourishing in sports participants is poorly understood. The purpose of this study was to i) evaluate HRQoL (physical and mental-components) and flourishing in recreational and elite and current and former cricketers; ii) determine the effect of resilience, playing-standard, and playing status on HRQoL and flourishing in cricketers.

**Methods:**

The Cricket Health and Wellbeing Study (*n* = 2598 current and former cricketers, aged ≥18 years) collected cross-sectional questionnaire data including the Flourishing Scale, Short Form-8 (physical (PCS) and mental (MCS) component scores), resilience (European Social Survey), highest standard-of-play, and playing status. Multivariable linear regressions with fractional polynomials were utilised, adjusted for age, gender, total cricket-seasons, comorbidity, ≥ 4-week time-loss injury, and orthopaedic surgery.

**Results:**

Two thousand two hundred eighty individuals (aged (mean (SD)) 51.7(14.7) years, 61% played recreationally, 37% former cricketers) were included in analyses. The median (IQR) PCS was 51.4(46.9–55.9), MCS was 54.3(50.0–58.6), and Flourishing Scale score was 48 [ (1–7)] .Greater resilience was associated with better PCS (effect (95% CI) 1.41(0.70–2.11)), MCS (4.78(4.09–5.48)), and flourishing (2.07(2.55–3.59)) compared to less resilience. Playing standard was not associated with HRQoL. Playing at an elite standard was associated with greater flourishing (1.21(0.68, 1.73)), compared with playing recreationally. Current cricket participation was associated with better PCS (3.61(2.92–4.30)) and flourishing scores (0.53(0.02–1.04)), compared to former cricket participation.

**Conclusion:**

Cricketers reported high levels of mental-components of HRQoL and flourishing, and this was similar in recreational, elite, current and former cricketers. Current cricket participation and a higher standard-of-play was associated with greater flourishing. Current cricket participation was also associated with better PCS, however playing-status was not related to MCS. Further research is needed to understand if cricket participation may have psychological benefits that persist beyond cricket retirement.

## Introduction

Over 30% of adults globally do not meet physical activity recommendations [8]. This is a great concern, considering physical inactivity increases the risk of cardiovascular disease, diabetes, and early mortality [9], escalating the overall disease and healthcare burden [10, 11]. To counteract the adverse health effects of physical inactivity, lifelong physical activity participation is advocated [11]. One avenue to promote physical activity is through sport participation [12]. Sport participation results in greater physical activity adherence and participation across the lifespan [13, 14]. One sport with worldwide participation is cricket [15]. Cricket is played at all ages and ability levels, with 104 countries being members of the International Cricket Council [15]. Playing cricket can enhance body composition, bone mineral density, muscle mass, and cardiovascular fitness [16–18]. However, despite many positive attributes to cricket participation, there can be adverse health effects including increased injury risk [19–21], subsequent joint pain and osteoarthritis [22, 23], with potential to negatively impact health-related quality of life (HRQoL). Investigation into physical and mental components of HRQoL in both current and former cricketers of all playing standards, may provide important information for assessing the appropriateness of cricket as an avenue to promote health across the lifespan.

Health-related quality of life is a multidimensional concept, incorporating multiple interrelated themes including physical function, social function, general health, energy, and mental health [24]. The most common instrument used to assess HRQoL is the RAND 36-Item Health Survey (SF-36) and its amended versions [25, 26], these instruments provide summary scores differentiating the mental and physical-components of HRQoL [27]. Within the general population, mental and physical-components of HRQoL are typically positively correlated [28]. In contrast, a recent systematic review and meta-analysis found that former athletes, who reported impaired physical-components of HRQoL compared to the general population, surprisingly reported better mental-components of HRQoL than the general population [29]. Impaired physical components of HRQoL in former athletes may be due to decreased physical function and osteoarthritis, secondary to joint injury [30, 31]. The high mental-components of HRQoL in former athletes with impaired physical-components of HRQoL, could be related to the psychological strengths (such as resilience) that are common amongst successful athletes [32], however no studies have investigated this [29]. Evaluating HRQoL in former sport participants is important, since it is unclear whether the positive impacts of sport on HRQoL [33, 34] extend beyond participation in sport, or whether strategies need to be developed to enhance HRQoL after retirement from sport. Notably, the only studies that have investigated mental and physical components of HRQoL in former sport participants have done so in former Professional American Football players (2 studies), or former Division 1 USA collegiate athletes (5 studies) [29]. The systematic review by Filbay et al. [29] highlights the need for research on HRQoL within other sports, particularly in non-contact sports, in sport participants outside of the USA, and within recreational sport.

People can participate in sport at all standards-of-play, ranging from recreational (social) standards to elite sport participation [35, 36]. However the majority of research has focused on elite athletes [37, 38], providing an incomplete picture of the effects of sports participation for the majority of individuals who take part in sport recreationally. Elite and recreational sport has different physical and psychological demands [39–41], which may have implications for HRQoL. For example, elite athletes are exposed to greater competitive stressors and a higher injury rate compared to recreational sport participants [39, 40, 42]. This could have negative impacts on HRQoL, or on the contrary, such challenges could build resilience with potential positive impacts on HRQoL. Due to a scarcity of research in recreational sport, the relationship between the standard of sports participation and HRQoL in current and former sport participants remains unclear.

Flourishing encompasses a holistic representation of physical, psychological, and social health [43, 44]. The construct of flourishing includes domains related to life purpose and meaning, which are important contributors to overall wellbeing that are not captured in HRQoL instruments [43]. Flourishing is considered the highest level of health, and has positive individual and societal impacts [45–48]. People who are flourishing have improved learning ability, greater work productivity, lower healthcare costs, and longer life expectancy compared to non-flourishers [46–48]. Flourishers are also considered more optimistic, have more meaning in their lives, and are able to better relate to their peers, improving overall life satisfaction and wellbeing [46, 49, 50]. While flourishing is an important holistic construct, only general population research has been performed [46, 49, 50], with no research in current or former sport participants. Consequently, there is little understanding of the relationship between sport participation and flourishing, and factors that affect flourishing in current and former sport participants have not been investigated.

The purpose of this study was to i) evaluate HRQoL (physical and mental-components) and flourishing in recreational and elite, and current and former cricketers; ii) determine the effect of resilience, playing-standard, and playing status on HRQoL and flourishing in cricketers. It is hypothesized that greater resilience, having played cricket at an elite standard (compared to recreationally), and current cricket participation (compared to former cricket participation) will be associated with greater HRQoL and flourishing.

## Methods

### Study design

A cross-sectional questionnaire was employed for the Cricket Health and Wellbeing Study. This study was approved by the NHS Health Research Authority (NRES), London Stanmore Research Ethics Committee (REC 15/LO/1274).

### Participants and recruitment

The England and Wales Cricket Board national database was used in March 2017 to recruit current and former cricketers from all standards-of-play. A total of 28,152 current and former cricketers were invited by email to participate. Two thousand five hundred ninety-eight cricketers deemed themselves to be eligible, responded and consented to participate. Eligibility criteria for this study consisted of having played ≥1 cricket season, age ≥ 18 years old, no history of memory impairment, and completed study outcomes (the SF-8 (HRQoL) and The Flourishing Scale).

### Questionnaire design

In collaboration with the England and Wales Cricket Board, the questionnaire was created and was then piloted on a group of current and former cricketers. The questionnaire was designed to evaluate five aspects of health and wellbeing: i. cricket-related injury; ii. joint pain and osteoarthritis; iii. General health and disease prevalence; iv. physical activity; v. resilience, quality of life and flourishing (the focus of this study). Participant data was inputted into an encrypted database. RedCap® software [51] was used, and participants could save their progress, and return to complete at a later time. The questionnaire gathered data on age, sex, body mass index (BMI), other sports played, number of cricket seasons played, years since retirement from cricket, number of joints injured, number of orthopaedic surgeries, persistent joint pain, physician diagnosed osteoarthritis, history of playing sport while injured, HRQoL, and flourishing.

#### Outcomes

##### Health related quality of life

HRQoL was captured through the Short Form 8 (SF-8) [25]. The SF-8 is a modified form of the SF-36 1.0 [27]. HRQoL is assessed on a 0 to 100 scale, with 0 representing full disability, and 100 representing no disability [1]. The SF-8 is comprised of the Physical Component Score (PCS) and Mental Component Score (MCS), which both have high reliability in general population samples [2]. The PCS and MCS are calculated using a normative based algorithm, using data from a sample of the 1998 United States general population. A mean of 50 is calculated as the normative average, and group mean scores below 47 are interpreted as below the average range of the general population [2]. Two points has been determined as the minimum detectable difference for the PCS in a lower extremity osteoarthritis sample [3], the minimum detectable difference has not been calculated for the MCS. Three to five points has been estimated as the minimum clinically important difference for the PCS and MCS for use in the general population [4].

##### Flourishing

Flourishing was assessed using The Flourishing Scale [44]. The flourishing scale is an 8-item survey that measures perceived success in interpersonal relationships, self-esteem, purpose, competence, and optimism [44, 45]. Each question is scored on a scale from 1 (strong disagreement) to 7 (strong agreement), with scores ranging from 8 (strong disagreement on all items) to 56 (strong agreement on all items). A higher score is considered greater flourishing [44]. The Flourishing Scale has been found to have high reliability (0.83 to 0.87) [44, 45] and validity in university students [44, 45] and in adults from a variety of socioeconomic backgrounds, cultural origins, and age ranges [49].

#### Explanatory variables

##### Resilience

Resilience was quantified using a line item from the European Social Survey, ‘*Please tell me to what extent you agree or disagree with this statement: When things go wrong in my life, it generally takes me a long time to get back to normal.’* This item question is part of a larger survey designed to investigate people’s comprehensive psychological wellbeing. This questionnaire was developed over two waves (2006 and 2012) of the European Social Survey. Over the two waves, based on the psychometric factor analysis, items were replaced for overall model best fit. Resilience was maintained for both waves. Response options were on a Likert scale of 1 (Agree strongly), 2 (Agree), 3 (Neither agree or disagree), 4 (Disagree) and 5 (Disagree strongly) [5]. Responses were stratified into resilient (score of 4 or 5) and not resilient (score of 1 to 3).

##### Standard-of-play

Standard-of-play was measured by the following question, ‘*What was the highest standard of cricket that you played for at least one season*?’ Response options included: international; county/premier league; academy or county age group; university; school; village or social; don’t know. Participants were stratified into recreational (university, school, village or social) and elite (international or county/premier league, academy or county age group). ‘*Don’t know*’ responses were excluded from analyses.

##### Playing status

Participants were asked to report their playing status. Response options included: ‘*Currently playing cricket*,’ ‘*No longer playing cricket*,’ and ‘*Plan to return to cricket*.’ Participants who reported that they ‘*Plan to return to cricket*’ were excluded from the analyses due to these participants could represent cricketers who are not currently playing for varied reasons, like current injury, travel, out of season, or illness, as this would potentially confound the results.

### Covariates

Covariates were identified following discussion, literature review, and clinical reasoning. Covariates included age, gender, number of cricket seasons played, presence of a comorbidity, history of orthopaedic surgery, history of a cricket-related joint injury resulting in ≥4 weeks of reduced participation in sport, training or exercise. For the purpose of this study, comorbidities was defined as a history of diabetes, stroke, skin cancer, or other cancer, due to their potential effect on HRQoL and flourishing [6, 7, 52, 53]. This data was converted to a binary variable (history of diabetes, stroke, skin cancer, or other cancer vs. no history of diabetes, stroke, skin cancer, or other cancer). Persistent pain was assessed through the following question, *‘Have you had pain in your [hip/groin, knee, ankle, shoulder, hand/finger, spine/back, other joint] on most days of the last month?’* A history of cricket-related joint injury was assessed with the following question, ‘*Have you ever had any cricket related injuries leading to more than 4 weeks of reduced participation in exercise, training or sport?*’ Participants were stratified into never sustained a joint injury (0), and sustained a joint injury (1). Number of orthopaedic surgeries were assessed by asking the following question, ‘*Have you ever had orthopaedic surgery (including bone, ligament or joint surgery)?*’ Participants were stratified into never had an orthopaedic surgery (0), and had an orthopaedic surgery (1).

### Statistical analyses

Data was not assumed to be linear; thus, they were modelled with fractional polynomials. Multivariable linear regressions with fractional polynomials were performed to assess the effect of resilience, standard-of-play and playing status on HRQoL and flourishing. Unadjusted and adjusted coefficients and 95% confidence intervals (95% CI) were calculated. Due to only 3% of all participants were female, sensitivity analyses were performed. HRQoL and flourishing scores were split for male and females, and then Mann-Whitney U tests were performed to assess for HRQoL and flourishing differences. Further, regression analyses were performed, consisting of only males, to evaluate the effect of resilience, standard-of-play and playing status on HRQoL and flourishing. All assumptions for fractional polynomial regression were evaluated and satisfied [54]. Resilience regression models were adjusted for age, gender, number of cricket seasons played, presence of comorbidities, history of ≥4 week time loss joint injury, and history of orthopaedic surgery. Standard-of-play and playing status regression models were adjusted for gender, number of cricket seasons played, history of ≥4-week time loss joint injury, and history of orthopaedic surgery.

Prior to analyses, all data were assessed for missingness. Due to the low percentage of missing data (Flourishing: 7.6%, MCS: 6.5% PCS; 6.5%, Resilience: 5.7%, Age < 1%) complete case analyses were performed. All analyses were performed in R version 3.5.1 (R Core Team (2013). R: A language and environment for statistical computing. R Foundation for Statistical Computing, Vienna, Austria. URL http://www.R-project.org/), using the dplyr package [55] for cleaning and coding, the naniar package for missingness assessment [56], and the mfp package for fractional polynomial regression [57].

## Results

A total of 318 participants from the Cricketers Health and Wellbeing Study were not eligible to take part in this study (did not provide information regarding age or total seasons played to determine eligibility (*n* = 91); played < 1 year of cricket (*n* = 2); < 18 years old (*n* = 7); reported history of memory impairment (*n* = 23); did not complete the SF-8 or The Flourishing Scale (*n* = 195)).

A total of 2280 cricketers (aged mean 51.7 SD 14.7 years, played a median of 30 IQR 18 to 42 seasons, 39% played at an elite level) met the eligibility criteria and were included in the analyses (Table 1). 47% had sustained a joint injury that resulted in a time loss injury of ≥4 weeks, 35% had undergone at least one orthopaedic surgery, and 62% were resilient. The median PCS score was 51.4 (IQR 46.5 to 55.9), the MCS score was 54.3 (IQR 50.2 to 58.5), and the median flourishing score was 48 (IQR 45 to 52) (Table 2). Elite cricketers reported a median PCS score of 51.9 (IQR 47.2 to 56.6), a MCS score of 55.0 (IQR 50.7 to 59.4), and a flourishing score of 49 (IQR 46 to 53). Recreational cricketers reported a PCS score of 51.3 (IQR 47.0 to 56.0), a MCS score of 53.7 (IQR 49.5 to 58.0), and a flourishing score of 48 (IQR 45 to 52).

**Table 1:** Participant characteristics

	All Cricketers (*n* = 2280)	Elite Cricketers (*n* = 872)	Recreational Cricketers (*n* = 1363)	Current Cricketers (*n* = 1425)	Former Cricketers (*n* = 855)
Age (years)	51.7 (SD 14.7)	50.7 (SD 15.6)	52.3 (SD 14.2)	46.3 (SD 13.8)	61.0 (SD 11.2)
Sex					
Male	2215 (97%)	842 (97%)	1317 (97%)	1296 (97%)	824 (97%)
Female	65 (3%)	27 (3%)	35 (3%)	39 (3%)	24 (3%)
BMI (kg/m2)	27.2 (IQR 24.6–29.9)	27.2 (IQR 24.6–29.8)	27.3 (IQR 24.7–29.9)	26.8 (IQR 24.4–29.3)	27.9 (IQR 25.3–30.6)
Cricket seasons played	30 (IQR 18–42)	32 (IQR 21–43)	27 (IQR 15–42)	28 (IQR 16–41)	32 (IQR 22–42)
Presence of comorbidity					
No	1779 (84%)	670 (83%)	1065 (85%)	1148 (91%)	549 (71%)
Yes	339 (16%)	133 (17%)	194 (15%)	113 (9%)	221 (29%)
Joints injured					
0	1195 (53%)	389 (45%)	786 (59%)	662 (50%)	476 (57%)
1+	1058 (47%)	469 (55%)	555 (41%)	662 (50%)	359 (43%)
Orthopaedic surgeries					
0	1469 (65%)	530 (61%)	904 (67%)	890 (67%)	517 (61%)
1+	800 (35%)	334 (39%)	445 (33%)	437 (33%)	328 (39%)
Persistent joint pain					
No	1305 (60%)	462 (56%)	809 (63%)	814 (65%)	431 (52%)
Yes	872 (40%)	364 (44%)	486 (37%)	451 (35%)	390 (48%)
Physician diagnosed osteoarthritis					
No	1598 (72%)	571 (69%)	986 (75%)	1012 (78%)	513 (63%)
Yes	611 (28%)	262 (31%)	337 (25%)	286 (33%)	305 (37%)
Standard of play^a^					
Elite	872 (39%)			521 (40%)	321 (38%)
Recreational	1363 (61%)			783 (60%)	517 (62%)
Resilience					
Resilient	1334 (62%)	524 (64%)	781 (61%)	801 (63%)	481 (61%)
Not Resilient	814 (38%)	292 (36%)	499 (39%)	466 (37%)	301 (39%)

*SD* standard deviation, *IQR* inter quartile range, *BMI* body mass index

^a^Participants were stratified into recreational (university, school, village or social) and elite (international or county/premier league, academy or county age group)

**Table 2:** Health related quality of life and flourishing in elite and recreational, current and former cricketers

	All Cricketers (*n* = 1978)	Elite Cricketers (*n* = 872)	Recreational Cricketers (*n* = 1363)	Current Cricketers (*n* = 1425)	Former Cricketers (*n* = 855)
SF-8 Domain Scores					
General Health Perceptions	46.4 (IQR 43.2–49.6)	46.4 (IQR43.2–49.6)	46.4 (IQR 43.2–49.6)	46.3 (IQR 43.2–49.6)	46.4 (IQR 39.2–53.6)
Physical Function	48.3 (IQR 45.5–51.2)	48.3 (IQR 45.5–51.2)	48.3 (IQR 45.5–51.2)	54.1 (IQR 51.3–57.0)	48.3 (IQR 41.3–55.3)
Bodily Pain	53.4 (IQR 50.6–56.3)	53.4 (IQR 50.6–56.3)	53.4 (IQR 50.6–56.3)	53.4 (IQR 50.6–56.3)	47.7 (IQR 41.1–54.4)
Physical Role Function	54.0 (IQR 50.5–57.6)	54.0 (IQR 50.5–57.8)	54.0 (IQR 50.5–57.8)	54.0 (IQR 50.5–57.8)	54.0 (IQR 50.5–57.8)
Emotional Role Function	52.4 (IQR 49.0–55.8)	52.4 (IQR 49.0–55.8)	52.4 (IQR 49.0–55.8)	52.4 (IQR 49.0–55.8)	52.4 (IQR 49.0–55.8)
Social Function	55.3 (IQR 52.4–58.2)	55.3 (IQR 52.4–58.2)	55.3 (IQR 52.4–58.2)	55.3 (IQR 52.4–58.2)	55.3 (IQR 52.4–58.2)
Vitality	55.6 (IQR 50.4–60.9)	55.6 (IQR 50.4–60.9)	55.6 (IQR 50.4–60.9)	55.6 (IQR 50.4–60.9)	55.6 (IQR 50.4–60.9)
Mental Health	49.6 (IQR 46.0–53.2)	56.8 (IQR 53.2–60.4)	49.6 (IQR 46.0–53.2)	49.6 (IQR 46.0–53.2)	56.8 (IQR 53.2–60.4)
SF-8 Component Scores					
Physical Component	51.4 (IQR 46.5–55.9)	51.9 (IQR 47.2–56.6)	51.3 (IQR 47.0–56.0)	52.4 (IQR 48.4–56.4)	49.6 (IQR 44.1–55.2)
Mental Component	54.3 (IQR 50.2–58.5)	55.0 (IQR 50.7–59.4)	53.7 (IQR 49.5–58.0)	53.8 (IQR 49.4–58.3)	54.9 (IQR 50.9–59.0)
Flourishing Total Score	48 (IQR 45–52)	49 (IQR 46–53)	48 (IQR 45–52)	48 (IQR 45–52)	48 (IQR 45–51)

### The effect of resilience, playing-standard, and playing status on HRQoL and flourishing in cricketers

After adjusting for covariates, cricketers that were resilient reported an estimated 1.41 (95% CI 0.70 to 2.11) points greater PCS, 4.78 (95% CI 4.09 to 5.48) points greater MCS, and 3.07 (95% CI 2.55 to 3.59) points greater flourishing scores, compared to cricketers that were not resilient (Table 3).

**Table 3:** The effect of resilience, playing-standard, and playing status on HRQoL and flourishing in cricketers

	Physical Component Score		Mental Component Score		Flourishing Score	
	Unadjusted Effect (95% CI)	Adjusted^a, b^ Effect (95% CI)	Unadjusted Effect (95% CI)	Adjusted^a, b^ Effect (95% CI)	Unadjusted Effect (95% CI)	Adjusted^a, b^ Effect (95% CI)
Resilient^d^ (*n* = 1334, 62%)	1.43 (0.72, 2.15), p < 0.0001	1.41 (0.70, 2.11), *p* < 0.0001	5.05 (4.38, 5.72), p < 0.0001	4.78 (4.09, 5.48), p < 0.0001	3.19 (2.70, 3.68), *p* < 0.0001	3.07 (2.55, 3.59) *p* < 0.0001
Not Resilient (*n* = 814, 38%)	*Reference Group*		*Reference Group*		*Reference Group*	
Elite^e^ (*n* = 872, 39%)	0.01 (−0.71, 0.72), *p* = 0.993	0.49 (−0.23, 1.22), *p* = 0.184	0.65 (− 0.06, 1.36), *p* = 0.074	0.27 (− 0.45, 0.99), *p* = 0.460	1.30 (0.79, 1.81), *p* < 0.0001	1.21 (0.68, 1.73) *p* < 0.0001
Recreational (*n* = 1363, 61%)	*Reference Group*		*Reference Group*		*Reference Group*	
Current^f^ (*n* = 1425, 63%)	3.57 (2.88, 4.26), p < 0.0001	3.61 (2.92, 4.30), p < 0.0001	−0.57 (−1.27, 0.12), *p* = 0.106	− 0.39 (− 1.09, 0.30), p = 0.270	0.39 (− 0.11, 0.89), *p* = 0.127	0.53 (0.02, 1.04), *p* = 0.042
Former (*n* = 855, 37%)	*Reference Group*		*Reference Group*		*Reference Group*	

^a^ Estimates for resilience are adjusted for age, gender (male = 0, female = 1), number of cricket seasons played, presence of comorbidities, history of ≥4 week time loss joint injury (no joints injured = 0, sustained a joint injury = 1), history of orthopaedic surgery (never had an orthopaedic surgery = 0, underwent orthopaedic surgery = 1)

^b^ Estimates for standard-of-play and playing status are adjusted for gender (male = 0, female = 1), number of cricket seasons played, history of ≥4-week time loss joint injury (no joints injured = 0, sustained a joint injury = 1), history of orthopaedic surgery (never had an orthopaedic surgery = 0, underwent orthopaedic surgery = 1)

^c^ All values are points on the Short-Form 8 Health Survey and Flourishing Scale

^d^ Resilience was defined as not resilient (0) and resilient (1)

^e^ Playing standard was defined as recreational (0) and elite (1)

^f^ Playing status was defined as former (0) and current (1)

^h^ SF-8: Short-Form 8 Health Survey; Physical Component Scores (PCS) were calculated using norm based scoring (population norm 50 SD 10, high scorer = better health-related quality of life); Mental Component Scores (MCS) were calculated using norm based scoring (population norm 50 SD 10, high scorer = better health-related quality of life)

^i^ The flourishing scale scores range from 8 (strong disagreement on all items) to 56 (strong agreement on all items). A higher score is considered greater flourishing

After adjusting for covariates, PCS and MCS scores were similar between elite and recreational cricketers. Playing at an elite standard was associated with greater flourishing scores compared with playing recreational cricket (1.21 (95% CI 0.68 to 1.73)) (Table 3).

Current cricket participation was associated with 3.61 (95% CI 2.92 to 4.30) points greater PCS and 0.53 (95% CI 0.02 to 1.04) points greater flourishing scores, compared with former cricket participation. Playing status was not related to MCS scores (Table 3).

### Sensitivity analyses

The male cricket mean age was 51.9 (SD 14.7) years, median BMI 27.3 (IQR 24.6 to 29.9), and median cricket seasons played were 30 (IQR 19 to 41) seasons. The female cricket mean age was 43.8 (SD 13.9) years, median BMI 24.4 (IQR 21.4 to 27.4), and median cricket seasons played were 9 (IQR 4 to 14) seasons. The male median PCS score was 51.4 (IQR 46.7 to 55.9), the MCS score was 54.3 (IQR 50.0 to 58.6), and the median flourishing score was 48 (IQR 45 to 52). The female median PCS score was 51.0 (IQR 46.0 to 58.3), the MCS score was 52.4 (IQR 46.6 to 58.3), and the median flourishing score was 48 (IQR 44 to 52). There were no statistical differences between male and female PCS (W = 64,354, *p* = 0.423), MCS (W = 65,098, *p* = 0.238), or flourishing scores (W = 57,674, *p* = 0.801). There were similar relationships between resilience, playing-standard, and playing status on HRQoL and flourishing in male cricketers as compared to all cricketers (Appendix).

## Discussion

### Summary

Compared to general population samples, elite, recreational, current and former cricketers reported similar physical-components of HRQoL, better mental-components of HRQoL and greater flourishing scores. Supporting the hypothesis, resilience was related to greater physical and mental-components of HRQoL and flourishing. Contrary to the hypothesis, playing cricket at an elite or recreational standard was not related to HRQoL; however, having played at an elite standard was related to greater flourishing compared with a recreational standard. Interestingly, playing status was not related to mental-components of HRQoL despite better physical-components of HRQoL (clinically meaningful difference) and better flourishing scores in current cricketers compared with former cricketers.

### Health-related quality of life

As described within the methods, the HRQoL normative scoring is calculated using a sample of the 1998 United States general population. This sample was composed of cricketers from the United Kingdom. While both countries speak English, and share similar cultures, there are cultural and time differences. Due to this, the potentiality for cultural measurement invariance exists when scoring and interpreting this data [58, 59]. Measurement invariance is the comparability of psychometric properties across cultures [60]. Studies have investigated the United Kingdom measurement invariance of the Short Form 36, and its many iterations [61–63]. While the reliability and psychometric properties are high, two studies are from the 1990’s [61, 62], and the other study is from the Wales population [63]. Thus, these results must be interpreted with caution since this sample was conducted in the 2017 and was composed of cricketers from the United Kingdom.

Elite, recreational, current, and former cricketers reported similar physical-components of HRQoL compared to the general population. These findings are in contrast to a recent systematic review, in which former athletes reported impaired physical-components of HRQoL compared to the general population [29]. However, the majority of sports included in this meta-analysis were contact or collision sports such as American football [29], which may result in greater physical impairment following retirement [64, 65]. While there are high injury rates in cricket [19–21], joint injuries may be less severe on average than those that occur in collision sports, and the positive physical contributions of cricket, such as strength [66], and physical fitness [18, 66], may potentially offset the physical impairments sustained due to sport participation.

In contrast, all cricketer subgroups reported clinically meaningful greater mental-components of HRQoL compared to the general population. This supports previous research in which athletes reported improved mental-components of HRQoL in comparison to the general population [29, 67]. ,Further, all cricketer subgroups reported improved emotional role function (functioning due to emotion), social function, and vitality compared to the general population. These findings give rise to the possibility that cricket participation may have psychological benefits that persist beyond cricket retirement, at all standards of play. It is also plausible that people with specific psychological strengths are drawn to competitive, team-based sports such as cricket. Further prospective research is needed to improve current understanding of the nature of the relationship between psychological strengths, mental-components of HRQoL and sport participation.

### Flourishing

All cricketer subgroups reported high flourishing scores, demonstrating that participants viewed themselves in positive terms in important areas of functioning [44]. While there are no current weighted normative values, comparisons can be made to different populations. Median flourishing scores in all cricketer subgroups ranged from 48 to 49, these are greater than those reported in a general population sample of Portuguese 25–60 year old adults (Flourishing Scale score: mean 43 SD 6) [45], a sample of 689 college students from Singapore and the United States (mean 45 SD 7, 37) and a sample of New Zealand 50–59 year old adults (mean 44 SD 8) [49]. There is no substantiated minimum detectable difference or clinically important difference for the Flourishing Scale, so we cannot yet ascertain whether these differences are clinically meaningful.

### Resilience

Cricketers that were resilient reported greater physical and mental-components of HRQoL and flourishing compared to cricketers that were not resilient. The association between greater resilience and better PCS scores was greater than the minimum detectable difference of two points [3], and the upper confidence interval was above the minimum clinically important difference of three points [4]; demonstrating a potentially clinically important difference [4]. Despite a positive association between greater resilience and HRQoL, cricketers reported equal resilience rates (62%) compared to the general United Kingdom population (62%) [5]. This suggests that there is potential to improve resilience amongst cricketers, and doing so, may have positive implications for HRQoL and flourishing.

One possible explanation for these findings, is that resilient cricketers with persistent joint pain (40% of all cricketers reported persistent joint pain) may be more likely to continue to perform physical tasks, compared to non-resilient cricketers (corresponding to greater HRQoL scores). This is supported by a randomised control trial, that found resilient patients with knee osteoarthritis, reported greater physical functioning compared to non-resilient patients with knee osteoarthritis [68]. Impaired physical function and pain can also contribute to greater athlete stress [69–71]. However, resilient athletes have been observed to have more positive thoughts and coping strategies during adversity and stressful situations [72]. Positive psychological outlook has been associated with improved HRQoL in sport populations [70, 73] and flourishing in the general population [74, 75]. One positive psychological construct within the flourishing scale is optimism [44]. Optimism is defined as a positive future outlook, which can help shape behaviour and positive outcomes [74]. Optimism and resilience are closely related, contributing to overall wellbeing [76, 77]. Resilient cricketers may have more aptitude to bounce back from stress and trauma [76], contributing to improved mental-components of HRQoL and flourishing [72, 77].

### Playing standard

Although playing standard was not related to HRQoL, having played elite cricket was associated with an estimated one point greater flourishing score compared to having played recreationally. While there are no studies investigating minimum detectable or clinically important differences in the Flourishing Scale, a difference of one point may not be clinically meaningful. This was the first study to evaluate the relationship between different standards of sports participation, HRQoL and flourishing. Our results suggest that sport participants from higher and lower standards have similar HRQoL and flourishing. Long-term stressors and environment have been shown to build resilience in athletes [39, 70, 78]. Sport participation has many similar stressors at all standards-of-play, including competition, injury, and organizational pressures [39, 70]. These stressors are present at the youth level [79, 80], and are consistent throughout an athlete’s sporting career [81]. Positive group membership has also been shown to build psychological strengths [82]. Cricket is a team based sport, with positive social and group interactions [83]. Elite and recreational cricketers first played cricket at a median of 10 and 11 years old and played for a median of 32 and 27 seasons, demonstrating an early and prolonged exposure to cricket participation. The continued sport stresses sustained throughout the lifespan, and the social and group interactions that occur in cricket may have similar effects on HRQoL and flourishing at all standards-of-play.

### Playing status

Current cricket participation was associated with greater physical-components of HRQoL compared with former cricket participation (after adjusting for covariates including age, seasons played, joint injury and surgery), and this is likely to be clinically meaningful [4]. Current cricketers also reported improved function, pain, and physical role function (function in activities that relate to work or daily living) [4] compared to the general population. This is in contrast to former cricketers who reported impaired function and pain compared to the general population. A possible explanation for these discrepancies is that current cricketers are more physically active than former cricketers. Physical activity can reduce pain, improve function, and physical performance [84]. Higher physical activity levels is also associated with increased HRQoL [85]. Another consideration it that cricketers with pain, functional impairments or osteoarthritis may be more likely to cease cricket participation. In our cohort 36% of cricketers reported injury or chronic pain as the reason for ceasing cricket participation. However, despite the discrepancies in physical-components of HRQoL, playing status was not related to mental-components of HRQoL. Sport participation may provide opportunities to maintain or gain psychological strengths and effective strategies to cope with pain or functional impairments throughout the lifespan [70, 73]. Other explanations include improvements in self-esteem, social connections, and goal achievement [33, 86]. In our cohort, the majority of individuals reported that cricket positively contributed to their concentration (85%), social skills (94%) and quality of life (93%).

#### Future research

In order to expand on these findings, further research is required. Cricketers were found to exhibit increased mental-components of HRQoL and flourishing, and these relationships were present in both recreational and elite cricketers. Further, these relationships were maintained after ceasing to play cricket. Cricket is a potential physical activity intervention, that can be played at all ages and standards of play. Due to these findings, research is required to understand the efficacy of cricket as a physical activity intervention. Specifically, the contribution of cricket to mental-components of HRQoL, flourishing, and psychological strengths requires further investigation. Other potential research avenues include investigating if resilience is inherent or learned during cricketer participation, the role of other sports and physical activities in relation to resilience, and how cricket and other sport participation can affect HRQoL and flourishing.

### Strengths and potential limitations

This study provided detailed analyses of physical and mental-components of HRQoL, in cricketers from all standards and abilities. Resilience was captured through a single-item question derived from the European Social Survey. While this question was developed over two waves of the European Social Survey, this single-item measure has not been validated or assessed for reliability beyond this survey. Using a validated and reliable patient reported resilience outcome measure would allow for improved inferences. The cultural measurement invariance for the SF-8, and its iterations has not been investigated. This decreases the precision of the HRQoL scoring and interpretation. In this sample, cricketers could play other sports, and perform other physical activities, which could affect HRQoL, flourishing, and resilience. Although the authors controlled for gender, number of cricket seasons played, history of ≥ 4-week time loss joint injury, and history of orthopaedic surgery in the multivariable analyses, playing other sports may still have a confounding effect upon the results. Due to the study recruitment methodology, it is not possible to determine a true response rate, decreasing the capability of this study to understand selection bias. Only 3% of all participants were female. This decreases the generalisability of these findings to female cricketers. Due to this, sensitivity analyses were performed. There were no differences in HRQoL or flourishing between males and females, and the only male analyses resulted in similar findings compared to the full analyses. However, future research is still required to evaluate a larger sample of female cricketers. This study only used self-reported outcome measures, which may cause common method bias. Finally, participants were asked to recall events that may have occurred in the past, which could create recall bias.

## Conclusion

Mental-components of HRQoL (i.e. emotional and social functioning, vitality and mental health) were better than the population average, in elite, recreational, current and former cricketers. Despite former cricketers reporting worse physical components of HRQoL (i.e. general health perceptions, physical function, pain) compared with current cricketers, mental components of HRQoL were comparably high in former and current cricketers. All cricketers reported high flourishing scores, although elite cricketers reported greater flourishing than recreational cricketers, this may not be clinically meaningful. Greater resilience was associated with better mental and physical components of HRQoL, and greater flourishing scores, in all cricketers. Further research is needed to understand if cricket participation at all playing standards, may be associated with positive mental impacts that persist beyond cricket participation. Additional research is required to determine whether these results are comparable in a larger sample of female cricket players, and if psychological benefits of sports participation can assist in reducing the personal burden of joint pain and osteoarthritis, which are common following prolonged sports participation.

## Data Availability

The datasets used and/or analysed during the current study are available from the corresponding author on reasonable request.

## Appendix

**Table 4:** The effect of resilience, playing-standard, and playing status on HRQoL and flourishing in male cricketers

	Physical Component Score		Mental Component Score		Flourishing Score	
	Unadjusted Effect (95% CI)	Adjusted^a, b^ Effect (95% CI)	Unadjusted Effect (95% CI)	Adjusted^a, b^ Effect (95% CI)	Unadjusted Effect (95% CI)	Adjusted^a, b^ Effect (95% CI)
Resilient^d^ (*n* = 1300, 63%)	1.36 (0.64, 2.08), *p* < 0.0001	1.40 (0.68, 2.12),*p* = 0.0001	4.98 (4.29, 5.66), p < 0.0001	4.73 (3.31, 6.15), p < 0.0001	3.23 (2.55, 3.59), p < 0.0001	3.07 (2.55, 3.59) p < 0.0001
Not Resilient (*n* = 766, 37%)	*Reference Group*		*Reference Group*		*Reference Group*	
Elite^e^ (*n* = 842, 39%)	0.01 (− 0.73, 0.73), *p* = 0.997	0.45 (−0.29, 1.19), *p* = 0.232	0.70 (− 0.02, 1.42), *p* = 0.058	0.31 (− 0.41, 1.04), *p* = 0.397	1.30 (0.79, 1.81), p < 0.0001	1.33 (0.81, 1.85) p < 0.0001
Recreational (*n* = 1317, 61%)	*Reference Group*		*Reference Group*		*Reference Group*	
Current^f^ (*n* = 1296, 59%)	3.59 (2.89, 4.29), p < 0.0001	3.61 (2.92, 4.29), p < 0.0001	−0.61 (− 1.09, 0.30), *p* = 0.270	−0.39 (− 1.10, 0.31), *p* = 0.274	0.34 (− 0.17, 0.85), *p* = 0.191	0.51 (− 0.01, 1.03), *p* = 0.053
Former (*n* = 915, 41%)	*Reference Group*		*Reference Group*		*Reference Group*	

^a^ Estimates for resilience are adjusted for age, gender (male = 0, female = 1), number of cricket seasons played, presence of comorbidities, history of ≥4 week time loss joint injury (no joints injured = 0, sustained a joint injury = 1), history of orthopaedic surgery (never had an orthopaedic surgery = 0, underwent orthopaedic surgery = 1)

^b^ Estimates for standard-of-play and playing status are adjusted for gender (male = 0, female = 1), number of cricket seasons played, history of ≥4-week time loss joint injury (no joints injured = 0, sustained a joint injury = 1), history of orthopaedic surgery (never had an orthopaedic surgery = 0, underwent orthopaedic surgery = 1)

^c^ All values are points on the Short-Form 8 Health Survey and Flourishing Scale

^d^ Resilience was defined as not resilient (0) and resilient (1)

^e^ Playing standard was defined as recreational (0) and elite (1)

^f^ Playing status was defined as former (0) and current (1)

^h^ SF-8: Short-Form 8 Health Survey; Physical Component Scores (PCS) were calculated using norm based scoring (population norm 50 SD 10, high scorer = better health-related quality of life); Mental Component Scores (MCS) were calculated using norm based scoring (population norm 50 SD 10, high scorer = better health-related quality of life)

^i^ The flourishing scale scores range from 8 (strong disagreement on all items) to 56 (strong agreement on all items). A higher score is considered greater flourishing

## Abbreviations

HRQoL: Health related quality of life
BMI: Body mass index
SF-8: Short form 8
PCS: Physical component score
MCS: Mental component score
